# How Does Herbal Medicine Treat Idiopathic Membranous Nephropathy?

**DOI:** 10.3389/fphar.2020.00994

**Published:** 2020-07-03

**Authors:** Zhendong Feng, Wenbin Liu, Han Xue Jiang, Haoran Dai, Chang Gao, Zhaocheng Dong, Yu Gao, Fei Liu, Zihan Zhang, Qihan Zhao, Lei Zhang, Baoli Liu

**Affiliations:** ^1^ Department of Nephropathy, Beijing Hospital of Traditional Chinese Medicine, Capital Medical University, Beijing, China; ^2^ Department of Nephropathy, Beijing Traditional Chinese Medicine Hospital Pinggu Hospital, Beijing, China; ^3^ Beijing University of Chinese Medicine, Beijing, China; ^4^ Shunyi Branch, Beijing Hospital of Traditional Chinese Medicine, Beijing, China

**Keywords:** chronic kidney disease, idiopathic membranous nephropathy, herbal medicine, therapy, pathogenesis

## Abstract

Idiopathic membranous nephropathy (IMN) has made increasing progress in mechanism and treatment research. Herbal medicine is gradually being accepted as an alternative therapy in treating IMN. However, the intervention mechanism of herbal medicine in the treatment of membranous nephropathy is still unclear. In this review, we summarize some achievements of herb medicine in treating IMN and discuss the research direction of herb in IMN. Finally, we propose the dilemma about the study on the treatment of IMN with herb medicine. We hope that this article can bring some thoughts for clinical and scientific researchers on the treatment of IMN with herb medicine.

## Introduction

Chronic kidney disease (CKD) is becoming an increasing public health problem all over the world because it is associated with an increased risk of cardiovascular disease and mortality (Webster et al., 2017; Cernaro et al., 2019; Wu et al., 2019; Mantovani and Chiara, 2020; Paolo et al., 2020). As one of the main types of CKD, membranous nephropathy is a glomerular disease characterized by diffuse thickening of the basement membrane, subepithelial deposition of immune complexes, and granuloid-like deposition of IgG along capillary loops by immunofluorescence (Borza, 2016). According to its etiology, it can be divided into idiopathic membranous nephropathy (IMN) and secondary membranous nephropathy (SMN), among which the cause is not known called as IMN, and secondary diseases caused by other diseases are often called SMN, such as hepatitis b, SLE and other diseases (Couser, 2017). Currently, IMN has become a common cause of nephrotic syndrome in adults, with an increasing proportion of primary glomerular diseases (Cattran and Brenchley, 2017; Li et al., 2018). However, there is a lack of treatment. Currently, immunosuppressive agents are often used to treat IMN, but it has the dilemma of large side effects and high recurrence rate. Cyclosporine-treated patients had a recurrence rate of approximately 43%. After discontinuation of tacrolimus, 9 of 19 (47%) patients in the tacrolimus group relapsed again at 18 months (Praga et al., 2007; van de Logt et al., 2016). In recent years, the use of rituximab and other drugs has made great progress in the treatment of IMN. Rituximab is being recommended as a first-line drug, but its high cost limits its clinical application (Trivin-Avillach and Beck, 2019). However, Herbal medicines have long been used in the clinic two thousand years ago and have been considered an alternative therapy for the treatment of various diseases, including the prevention and treatment of CKD (Chen et al., 2018a; Chen et al., 2018b; Ma et al., 2018; Parveen et al., 2018; Chen et al., 2019a; Jing and Jin, 2020). In recent years, herb medicine in the treatment of IMN has achieved some success, In this article, we have summarized the clinical and mechanism research of herb medicine for IMN and hope to bring new thinking to the treatment of IMN.

## Effect of Herb Medicine on IMN

Mounting evidence demonstrated that the intervention mechanisms of herbal medicines on CKD were associated with renin–angiotensin system, inflammation and oxidative stress, aryl hydrocarbon receptor, transforming growth factor β (TGF-β)/Smad signaling, Wnt/β-catenin signaling pathways (Chen et al., 2019b; Chen et al., 2019c; Feng et al., 2020; Hu et al., 2020). In addition, herb medicine were used for the treatment of CKD by modulating the metabolic disorders such as amino acid metabolism and lipid metabolism (Zhang et al., 2018; Miao et al., 2020). A number of studies have demonstrated that herbal medicine could be used for the treatment of IMN (Xu et al., 2015; Shi et al., 2018; Wang et al., 2018c; Jin et al., 2020). In **Table 1**, we summarized the reports on the treatment of IMN with herbal medicine. Earlier, a case report reported that Astragalus membranaceus treated a 77-year-old woman with IMN and achieved clinical remission without using immunosuppressive agents (Ahmed et al., 2007). The efficacy and safety of Shenqi particle were assessed by using patients with IMN based on a multicenter randomized controlled clinical trial. The findings demonstrated that Shenqi particle was a promising alternative therapy for adults with IMN and nephrotic syndrome (Chen et al., 2013). In addition, it has been reported that 15 patients who fail to immunosuppressive therapy treated with Jianpiqushifang, and found that 80% of the patients were able to achieve clinical remission, and no obvious adverse reactions were found after 1 year of follow-up (Shi et al., 2018). Recently, the combination of tripterygium wilfordii multiglycosides and prednisone is considered as an effective and safe therapy for IMN. Shanshan Liu et al. found the probability of remission was similar for both the tripterygium wilfordii multiglycosides and tacrolimus group. Herb medicine also plays a role in improving the efficacy of immunosuppressants, wuzhi capsule can markedly improve the blood concentration of FK506 inpatients with IMN (Zhang et al., 2019). These studies have shown that herbal medicine can effectively treat IMN and relieve proteinuria, but the mechanism is still unclear.

**Table 1:** Chinese medicine treats membranous nephropathy.

Chinese medicine/prescription	Major herb medicine composition	Method	Primary outcome measure	References
Astragalus membranaceus		Case reports		(Ahmed et al., 2007)
wuzhi capsule	Deoxyschizandrin	Clinical observation	blood concentration of FK506	(Zhang et al., 2019)
Tripterygium glycosides		a prospective cohort study	remission rate	(Liu et al., 2015)
Jian Pi Qu Shi Formula	Astragalus membranaceus Poria cocos Codonopsis pilosula	Patients with IMN/retrospective study	complete remission/partial remission	(Shi et al., 2018)
Shenqi Particle	Astragalus membranaceus Poria cocos Atractylodes chinensis	Patients with IMN/randomized, controlled clinical trial	complete remission/partial remission	(Chen et al., 2013)

## Study on Mechanism of Herb Medicine Treatment of IMN

Most of the studies on the mechanism of herb medicine treatment of IMN focused on animal experiments, aiming to explain the mechanism of herb medicine treatment of IMN. Astragalus is one of the traditional Chinese medicines commonly used in the treatment of IMN. Rong Zheng et al. found that Astragaloside IV can attenuates complement membranous attack complex induced podocyte injury through decreasing the expression of extracellular regulated protein kinases (ERK) (Zheng et al., 2012). More than that, sanqi oral which mainly include Radix Astragalus membranaceus and Radix Notoginsen not only decrease inflammatory factors by inhibiting the level of NF-κB, but also reduce deposition of C3 and IgG in experimental rat model of membranous nephropathy induced by cationic Bovine Serum Albumin (C-BSA) (Tian et al., 2019). It is well know that NF-κB play an important regulator role in immune response (Bhatt and Ghosh, 2014). Recent studies have shown that NF-κB also participates in the pathogenesis of MN (Sutariya et al., 2017). Zhenwu Decoction can reduce the expression of pro-inflammatory factors by inhibiting the level of NF-κB and NLRP3,which will reduce cell apoptosis(Liu et al., 2019a). Another study suggests that zhenwu Decoction reduce the pro-oxidation ability of advanced glycation end (AGE) by down-regulating the expression of receptor AGE(RAGE) in podocyte, which reduce oxidative stress in podocyte (Wu et al., 2016). Interestingly, Zhenwu Decoction combined with Astragalus can also directly promote the expression of Bcl2 and inhibit the transcription of P53 to reduce cell apoptosis (Lu et al., 2020).

**Figure 1:**
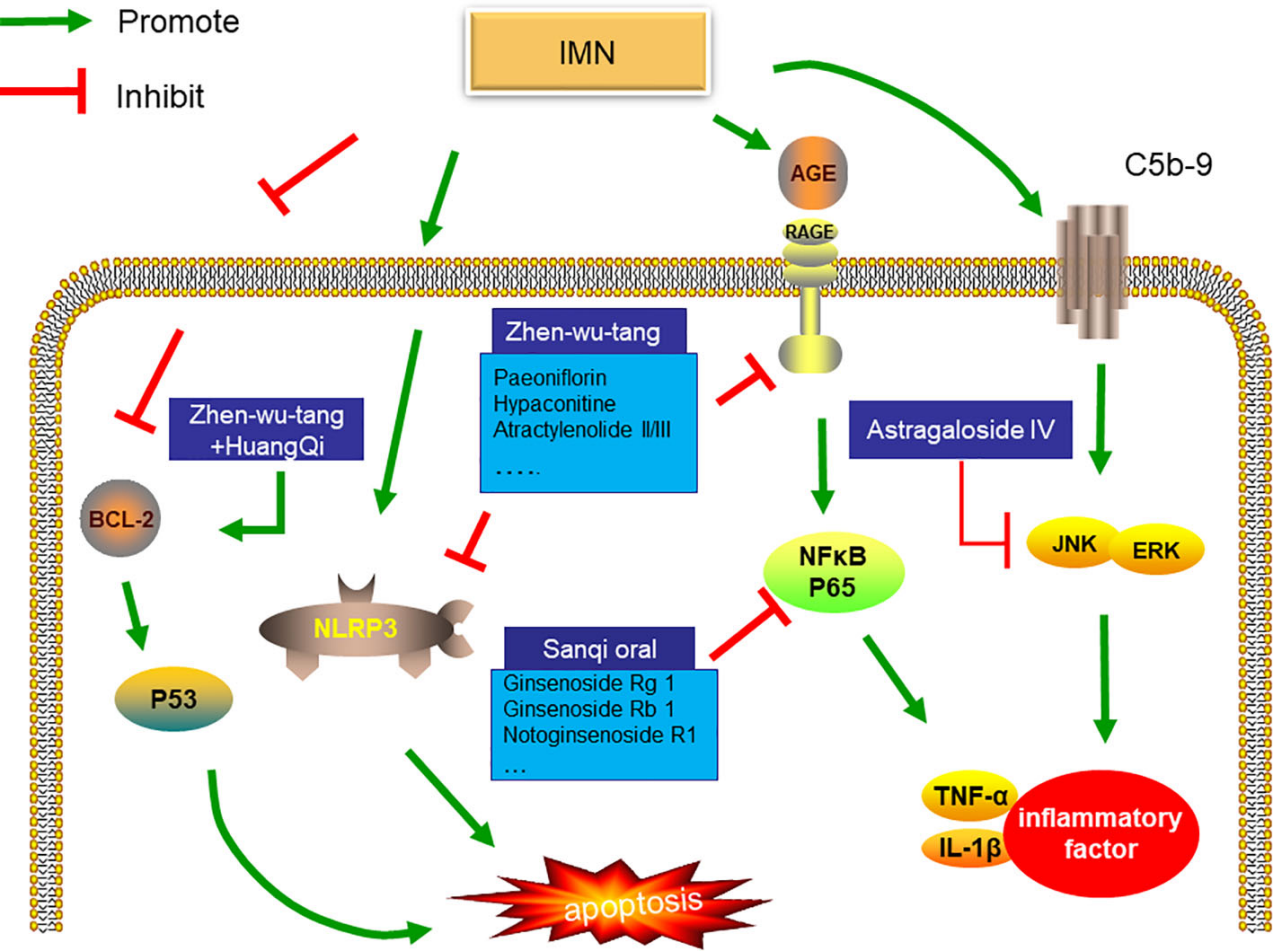
Mechanism of Chinese medicine treatment of IMN. Zhenwu decoction combined with Astragalus can also directly promote the expression of Bcl2 and inhibit the transcription of P53 to reduce cell apoptosis. In addition, zhenwu decoction reduce the pro-oxidation ability of AGE by down-regulating the expression of receptor AGE(RAGE) in podocyte, which reduce oxidative stress in podocyte. Not only that, Zhenwu decoction can reduce the expression of pro-inflammatory factors by inhibiting the level of NF-κB and NLRP3,which will reduce cell apoptosis sanqi oral which mainly include Radix Astragalus membranaceus and Radix Notoginsen not only decrease inflammatory factors by inhibiting the level of NF-κB, but also reduce deposition of C3 and IgG in experimental rat model of membranous nephropathy induced by cationic Bovine Serum Albumin (C-BSA). Astragaloside IV can attenuates complement membranous attack complex induced podocyte injury through decreasing the expression of ERK.

To sum up, prescriptions composed of the same herb medicine can also show different targets. For example, Zhenwu Decoction can regulate inflammatory factors and RAGE in podocyte. This may be the display of multiple targets of herb medicine. As shown in **Figure 1**, Existing research shows that the target of herbs is podocyte apoptosis and inflammation. However, it is worth noting that inflammatory infiltration is rarely noticed in the pathology of IMN. At present, the study of the mechanism of herb medicine treatment of IMN is focused on the kidney, other organs has not been observed. More recently, scholars have suggested that initial immune response may be in the lungs, not the kidneys in patients with IMN (Liu et al., 2019b), but Current research is still focused on the kidneys. The effect of herb on extrarenal organs still needs further study.

## The Research Direction of Herb Medicine Treatment IMN

### The Effect of Herb on Immunity in IMN

**Figure 2:**
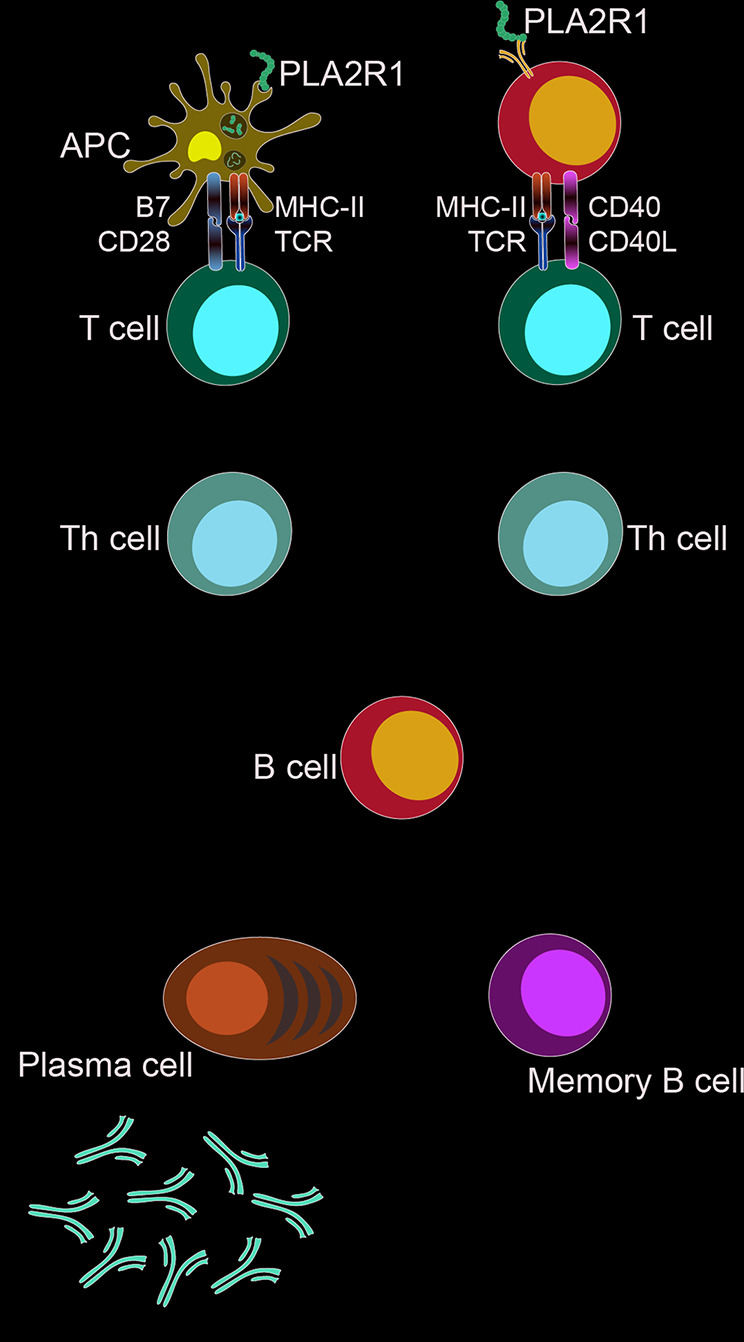
Target of drug action. The targets of immunosuppressive agents are different links in the immune response.

IMN is considered an autoimmune disease (Motavalli, et al., 2019). Since 2009, autoimmune antigens, such as PLA2R, THSD7A and Nell-1, have been gradually discovered, with 70–80% of IMN patients having PLA2R receptor positive (Beck et al., 2009; Tomas et al., 2014; Kao et al., 2015; Cui et al., 2017; Sethi et al., 2019). Autoantigens such as PLA2R are presented to T cells and B cells, activating an autoimmune response and then eventually produce autoantibodies that damage podocyte and induce proteinuria (Liu et al., 2019b; van de Logt et al., 2019). Studies have shown that the antibody level is closely related to the prognosis of IMN, and the lower the antibody level, the better prognosis of patients. In a cohort study, among 11 patients who were negative for serum anti-PLA2R1 and THSD7A and only received supportive treatment, 91% of patients experienced spontaneous remission and 7 patients achieved complete remission; no spontaneous remission when anti-PLA2R1 > = 40IU/ml, and high antibody levels had higher risk for developing ESRD (Hoxha et al., 2015; Diaz et al., 2019). Antibody levels are a reflection of the body’s immune activity, and modern medicine is precisely to treat IMN by suppressing the immune response, as shown in **Figure 2**. The preferred option in the KDIGO guidelines is a combination of hormones and alkylation agents, of which Cyclophosphamide (CTX) is recommended. Glucocorticoids can directly inhibit the expression of inflammatory factors and induce apoptosis of CD4^+^ CD8^+^ immature thymocytes, in addition, they induce the expression of IL-7 receptor a chain (IL-7Ra) in T cells to regulate immune (Shimba et al., 2018). However, prednisone treatment of IMN for 6 months did not show significant efficacy in a prospective randomized controlled trial (Cattran et al., 1989). Therefore, the use of glucocorticoids alone is not recommended, but it cannot be denied that long-term large-dose use of prednisone has potential effectiveness, due to the serious side effects of hormones, long-term use may not be a good choice. CTX significantly reduces the expression of serum IgG, IgM, IFN-γ, and IL-6 in animals (Fan et al., 2013). Studies have shown that CTX regulates cytokines through Dectin-1, TLR2 and TLR4 signaling pathways, changes Th1/Th2 balance in the body, and plays a role in regulating immunity (Yang et al., 2010; Fang et al., 2012; Logani et al., 2012). In the treatment of IMN, it mainly refers to cyclosporine and tacrolimus, of which CNI is KDIGO’s only initial replacement therapy program (Beck et al., 2013), cyclosporine can inhibit the NFAT “dephosphorylation” of T cells and inhibit the proliferation of T cells (Liddicoat and Lavelle, 2019). Twenty-five patients received tacrolimus (0.05 mg/kg/day) over 12 months with a 6-month taper, whereas 23 patients were in the control group. The probability of remission in the treatment group was 58, 82, and 94% after 6, 12, and 18 months but only 10, 24, and 35%, respectively in the control group (Praga et al., 2007). Rituximab (RTX) is a new scheme for the treatment of IMN and is now intended to be used clinically as a first-line regimen (Trivin-Avillach and Beck, 2019), RTX is a human-mouse chimeric monoclonal antibody that selectively targets the CD20 surface antigen of B lymphocytes and specifically inhibits the proliferation and activity of B lymphocytes (Parmentier et al., 2019). A randomized controlled study comparing rituximab to cyclosporine (Fervenza et al., 2019) showed that rituximab was not inferior to cyclosporine in inducing complete or partial remission of proteinuria at 12 months. Monthly maintenance of proteinuria is better than cyclosporine. Some scholars have tested the content of B cells in the serum of patients after rituximab treatment and found that the number of CD19^+^ B cells is close to 0 after eight days of rituximab treatment (Rosenzwajg et al., 2017). Recently, bevacizumab, a human IgG1-k monoclonal antibody B lymphocyte stimulation inhibitor binds to soluble human BLyS and inhibits its biological activity. In an open prospective study (Barrett et al., 2019), 11 PLA2R1-AB positive patients were enrolled and given bevacizumab monotherapy for 2 years. Nine participants achieved complete or partial response and it was found that bevacizumab can induce naive B cell reduction, trends towards increases in memory B cells, but the proportion of activated memory B cells was decreased.

The above results indicate that immunity plays an important role in the development of IMN, and suppressing the immune response is an important method to promote the remission of IMN. This may also be the mechanism of herb medicine treatment of IMN. Herb medicine commonly used in the treatment of IMN have the function of regulating immunity. *Astragalus membranaceus* is considered to be a natural immunomodulator with the ability to regulate phagocytosis of neutrophils and macrophages (Vetvicka and Vetvickova, 2014). Astragalosides (ASIs) is one of the main active components of Astragalus. There is increasing evidence that ASIs are reported to be experimentally used in two contrary immune-associated diseases. On the one hand, *Astragalus* *membranaceus* can enhance the proliferation of TB lymphocytes in LPS-stimulated splenocytes. On the other hand, ASIs can also inhibit the production of IL-17 and induce regulatory T cells. Play a protective role in autoimmune diseases is one of the main active components of *Astragalus membranaceus* (Qi et al., 2017). *Tripterygium Wilfordii* consists of more than 70 constituents, including diterpenes, triterpenes and glycosides, which plays an immunomodulating role by inhibiting T cell proliferation (Yang et al., 2013). Therefore, we believe that immunity is likely to be one of the mechanisms of herb in the treatment of IMN, or may be the main way. But now the research of traditional Chinese medicine treatment of IMN focuses on kidney and podocytes. Of course, we do not think that the therapeutic effects of herb are equivalent to immunosuppressants. We believe that herb medicine may play a role in promoting immune balance. Recent studies have shown that the proportion of Regulatory T cells (Treg) in peripheral blood of IMN patients is significantly lower than normal people, and the proportion of Th2 and Th17 cells is increased (Rosenzwajg et al., 2017). So it is necessary to pay attention to the regulation of IMN on immunity.

### Anti-Inflammatory and IMN

Antigen is the initial stage of immunity, after the antigen disappears, the body’s immune activity will gradually return to calm (Marrack et al., 2010). The exposure of antigen is closely related to the internal environment of the body. Studies have shown that the binding of IMN patient serum IgG antibodies to PLA2R1, THSD7A or NELL-1 *in vitro* needs to be carried out under non-reducing (Beck et al., 2009; Tomas et al., 2014; Sethi et al., 2019). The oxidizing environment will cause higher PH value for the extracellularly conditions relative to the intracellular environment, which may result in a more extended conformation of human PLA2R1 (Dong et al., 2017; Liu et al., 2019b). The PH dependent conformational change of human PLA2R1 may lead to the exposure of internal domains, which would be recognized by the B cell. Soluble PLA2R1, or protein fragments about PLA2R1 is engulfed by APC cells and presented to T cells, which provides a second signal for B cell activation. Therefore, the extracellular environment plays an important role in epitopes and autoimmune response (Ancian et al., 1995; van de Logt et al., 2019). For example, PM2.5 is closely related to the occurrence of IMN (Xu et al., 2016). PM2.5 is an important cause of chronic inflammation.

To sum up, it is a reasonable inference that improving the inflammatory environment of the body will help reduce the exposure of antigens, while Traditional Chinese medicine has definite anti-inflammatory and antioxidant effects.

*Astragalus membranaceus* is one of the drugs commonly used to treat membranous nephropathy. *Astragalus membranaceus* has significant antioxidant activity. Flavonoids *Astragalus membranaceus* are the main active antioxidants. They have significant antioxidant activity against superoxide anion and play an important role in heart and liver diseases (Fu et al., 2014). Previous studies have shown that *Astragalus membranaceus* can regulate nuclear factor-erythroid-2-related factor 2 (Nrf2) signaling pathway, inhibit p38 MAPK, nuclear factor-kappa B (NF-κB), and toll-like receptor mediated pathway in a variety of cells (Zhong et al., 2015). *Tripterygium Wilfordii* has also been reported to upregulate the Cytokine IL-37 through ERK1/2 and p38 MAPK Signal Pathways and decrease inflammatory by inhibiting prostaglandin E2 production (Maekawa et al., 1999; Wang et al., 2017), *Triptolide*, the main active ingredient in Tripterygium glycosides, can inhibit MMP-2 and MMP-9 expression and maintains Redox Balance on rheumatoid arthritis (Xie et al., 2019). *Poria cocos* is a well-known medicinal mushroom and it possesses various pharmacological activities such as anti-tumor, anti-inflammatory, antioxidantive, diuretic, renoprotective and lipid-lowering effects (Wang et al., 2013; Chen et al., 2019d). Polysaccharide from *Poria cocos* not only has significant antioxidant stress scavenging ability of free radicals, but also can regulate the levels of IL-2 (Li et al., 2019). Improving inflammatory environment is the embodiment of the theory of Whole View in IMN, but the relation of anti-inflammatory and the remission of IMN are just ignored in the research about herb medicine. It is worthy of further study that herb promote IMN remission by inhibiting inflammation.

### Effect of Herb Medicine on Podocytes in IMN

Proteinuria is the result of impaired renal filtration function, and the decrease or disappearance of proteinuria is the result of filtration barrier repair. Glomerular filtration barrier (GFB) is a highly specified blood filtration boundary in kidney and consists of highly differentiated epithelial cells called podocyte, glomerular basement membrane (GBM), and fenestrated endothelium (Hosseiniyan Khatibi et al., 2020). Damage to any of the three layers will lead to proteinuria. The kidney tissue of patients with IMN can observe the fusion of podocytes and the deposition of immune complexes by electron microscopy and many kinds of evidence show that the damage of podocyte skeleton protein (Nangaku et al., 2005; Fogo et al., 2015). Therefore, podocyte damage is considered to be the main cause of proteinuria. It’s worth noting that podocytes is terminally differentiated cells, which means that podocyte damage is reversible in IMN, otherwise proteinuria will not disappear in the patient with remission. In some animal experiments, traditional Chinese medicine can improve the function of IMN podocytes, but the mechanism of herb medicine on human podocytes cannot be directly concluded, because the injury mechanism of human podocytes is different from that of rats. The hypothesis that the injury pathway of human podocyte is causative by antibody and complement is controversial. C5b-9 inserted into the podocyte membrane is transported inside the cell and squeezed into the urinary cavity, which is considered to be a dynamic sign of continuous immune injury (Ronco and Debiec, 2017). It’s confusing that serum anti-PLA2R1 antibodies and glomerular subepithelial immune complexes are mainly of IgG4 subtype (Qin et al., 2011; Hofstra et al., 2012), the ability of IgG4 to bind to C1Q is weak, and the ability to activate complement is insufficient. C1Q, which is a necessary molecule in the classical pathway to activate complement, staining is indeed lacking in kidney staining of IMN (Doi et al., 1984; Qin et al., 2011; Vidarsson et al., 2014; Koneczny, 2018). What’s more, it was found that eculizumab, which can block the formation of C5b-9, has no obvious effect for the treatment of IMN (Appel et al., 2002). In the animal model of IMN, the mechanism of complement damage to podocytes is also controversial. Despite the deposition of IgG under the epithelium, daily removal of serum complement factor C3 by cobra venom injection can still prevent proteinuria in Passive Heymann Nephritis (PHN) (Salant et al., 1980). However, there is some evidence that C5b-9 is not necessary for podocyte damage. It is reported that there is no significant difference in proteinuria between the PVG/C6- and PVG/C6 rats, and the proteinuria can appear in mice 3 days after injection of purified THSD7A antibody serum, but kidney tissue no C3 deposit was found in the staining (Tomas et al., 2016). In recent years, the theory that antibodies damage podocytes has also been proposed. Nicola et al. co-cultured the serum of mouse podocyte with THSD7A-positive patients *in vitro* and found that human-IgG combined with THSD7A on the surface of the mouse podocyte membrane showed significant skeleton rearrangement (Tomas et al., 2016). This may be related to the function of THSD7A. The binding of antibody to THSD7A inhibits the function of THSD7A, which may be involved in the adhesion function of cells, leading to podocyte damage (Tomas et al., 2016; Beck, 2017). PLA2R1 has a similar function. It is reported that M-PLA2R1 promotes the interaction between integrin β1 and collagen-I through the extracellular part to maintain podocyte stability (Watanabe et al., 2018). Therefore, the role of C5b-9 in podocyte injury is still controversial. This is debatable to explore the mechanism of herb medicine treatment of IMN in the C5b-9 pathogenic model. It is worth noting, immunosuppressive agents can promote the reduction of proteinuria in IMN, which seems to suggest that podocyte injury plays a secondary role. Some evidence suggests that steroids and immunosuppressants have some role in repairing podocyte (Faul et al., 2008; Guan et al., 2015; Li et al., 2015; Hosseiniyan Khatibi et al., 2020),but we don’t think that adjusting or repairing podocytes is the main role of immunosuppressants. It is reported that the response rate after 12 months of rituximab treatment was 60%, and the response rate after cyclosporine treatment was 52%. The remission rate in cyclosporine group is much lower than the rituximab group with time (Fervenza et al., 2019). Assuming that in membranous nephropathy, immunosuppressants have a dual role, which can both suppress immunity and improve podocyte function, the clinical remission rate should be higher than that of biological agents that only suppress immune effects, and the advantages should increase with time. However, existing studies have shown that rituximab is superior to cyclosporine in IMN. Of course, this is only a rough comparison, and it is also interfered by many factors. But it would be the fact improving podocyte function has taken a secondary role in IMN therapy. The mechanism that herb medicine improves podocytes may help us understand why one Chinese medicine can treat multiple kidney diseases, this may be because these herbs can improve podocyte injury, after all, podocyte injury is a common mechanism of renal proteinuria.

The above three research directions are not independent of each other. Traditional Chinese medicine may act on IMN through a variety of channels, and one prescription may contain anti-inflammatory and immunosuppressive effects. Different drugs or proportion of the prescription, the focus of treatment is different, which is also the embodiment of the individualization of traditional Chinese medicine. Immunosuppression alone or inflammatory therapy for IMN all have certain limitations. peripheral tolerance loss and T and B lymphocyte subgroup imbalance may be the result of chronic inflammation immunity education. At this point, simply suppressing inflammation may not be effective, after all,the immune system of such patients may also be abnormal.

## Difficulties in the Research of Chinese Medicine in IMN

**Figure 3:**
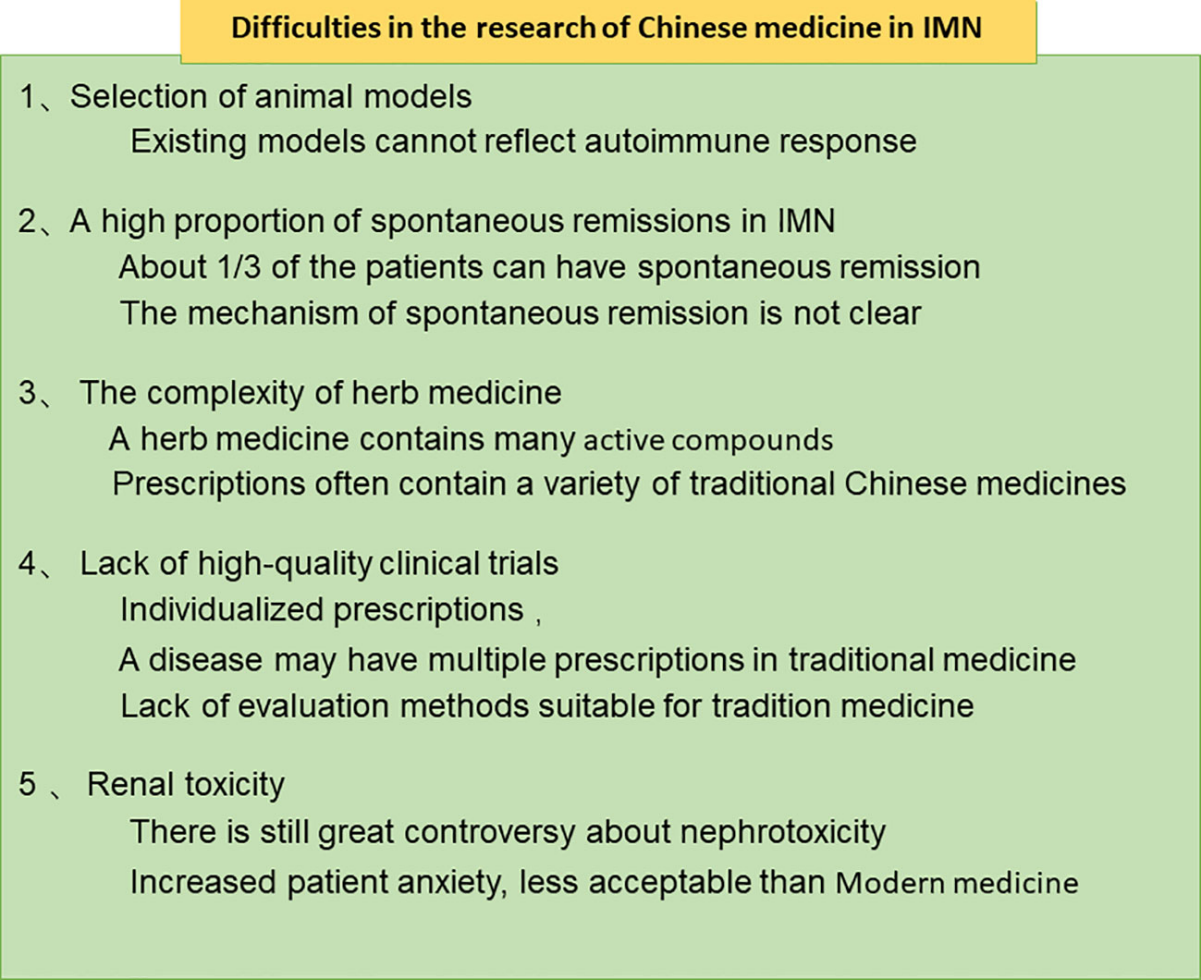
Difficulties in the research of Chinese medicine in IMN.

In recent years, some progress has been made in the treatment of IMN with herb medicine, but we still face many difficulties, as shown in **Figure 3**. Firstly, Selection of animal models. In 1959, Heymann prepared a classic membranous nephropathy model by using the brush border of rat renal tubules (Heymann et al., 1959). Heymann nephritis is a classic animal model for studying membranous nephropathy, which can be divided into active and passive Heymann nephritis. The active model uses homologous renal homogenate and complete Freund’s adjuvant to immunize rats through the intraperitoneal route, the passive model is to inject heterologous antibodies against rat kidney homogenate produced in sheep and rabbits into rats, which all show similar pathology to IMN (Heymann et al., 1959; Zanetti and Druet, 1980). The HN rat model is regarded as a classic animal model due to its high similarity in pathology to human IMN. But Heymann rats were created by injecting antibodies or antigens into the rats and could not simulate the process of an autoimmune response (Jiang et al., 2020), which hinders the study of herb on autoimmune response. Second, the complexity and diversity of the components of herb medicine. For example, there are more than 100 components of Radix Astragali (Fu et al., 2014). More difficult is that the clinical application of TCM is mostly compound medicine rather than single TCM, it is more difficult to analyze the active ingredients of traditional Chinese medicine. Third, Lack of large-scale clinical trials to prove the effectiveness of herb medicine, The percentage of each component in the formulas varies based on the symptoms and signs of individual patients. Individual components in each herbal prescription are then adjusted at each follow-up visit to achieve the maximal effects, This often leads to different prescriptions for everyone, which makes the design and operation of clinical trials difficult. Fourthly, the mechanism of spontaneous remission is unknown. High spontaneous remission rate is one of the characteristics of IMN, about 1/3 of the patients can have spontaneous remission (Tomas et al., 2017). Thus, the treatment of traditional Chinese medicine is often regarded as spontaneous remission that affects the evaluation of the efficacy of traditional Chinese medicine. In fact, attenuating oxidative stress or inflammatory environment all contributes to reduce antigen exposure and gradually suppress immune response, which may be the reason of spontaneous remission. Yet many factors in daily life may improve internal environment and promote remission. Diet, sleep, mood, and environment all play a role in regulating inflammation, gut flora, and immunity (Besedovsky et al., 2012; D’Acquisto, 2017). However, at present, there is a lack of cohort studies on daily exposure factors that cause self-antigens to become non-self in membranous nephropathy. Expect large cohort study on IMN predictors to reveal the truth about spontaneous remission. Fifth, evaluation of nephrotoxicity. The nephrotoxicity of traditional Chinese medicine is a controversial topic, how to understand and study nephrotoxicity is still an urgent problem to be solved.

## Concluding Remarks

In this article, we have summarized the current traditional Chinese medicine prescriptions and targets for IMN. According to the pathogenesis of IMN and other characteristics, we have suggested that immunity and inflammation may be another important mechanism of action for herb medicine to treat IMN. Finally, we pointed out the development dilemma of Chinese medicine in IMN. Overall, the intervention mechanisms of herbal medicines on IMN are still in its infancy compared with CKD-associated other diseases. We do not list all the mechanisms of herb in IMN. Herbal medicines on CKD could be used for treatment of membranous nephropathy by targeting mentioned-above associated-CKD mechanism. For example, RAS inhibitors could lower blood pressure and reduce glomerular capillary pressure, the leakage of proteinuria. It has been demonstrated that herbal medicines could effectively blocked RAS by simultaneously targeting multiple RAS components (Chen et al., 2018c; Wang et al., 2018a; Wang et al., 2018b). Hope this article can bring different thinking to the scholars.

## Author Contributions

ZF and WL collected most of the material for reviewing and wrote the main part of the review. HD and CG collected the rest of the material for reviewing. HJ, ZD, and YG wrote the rest part of the review. FL, LZ, ZZ, and QZ made the figures and tables. BL proposed the content of the review article. All authors contributed to the article and approved the submitted version.

## Funding

This work was supported by grants from the National Natural Science Foundation of China (no. 81673907,8197151276), National Key Research and Development Project (no. 2019YFC1709402), Natural Science Foundation of Beijing Municipality (no. 7182070).

## Conflict of Interest

The authors declare that the research was conducted in the absence of any commercial or financial relationships that could be construed as a potential conflict of interest.

The reviewer JL declared a shared affiliation, with no collaboration, with some of the authors, HJ, CG, ZD, FL, ZZ, to the handling editor at the time of the review.
